# Supplementary material for: Seasonal epiphytic microbial dynamics on grapevine leaves under biocontrol and copper fungicide treatments
Source: Sci Rep. 2020 Jan 20;10:681. doi: 10.1038/s41598-019-56741-z (PMC6971271; doi:10.1038/s41598-019-56741-z)
Supplement: Supplementary file 1 — Supplementary information. [file 41598_2019_56741_MOESM1_ESM.pdf]

Seasonal epiphytic microbial dynamics on grapevine leaves under biocontrol and copper fungicide treatments

Gobbi Alex[1], Kyrkou Ifigeneia[2], Filippi Elisa[2], Ellegaard-Jensen Lea[2], Hansen Lars Hestbjerg[1]

[1]Environmental Microbial Genomics (EMG), Plant and Environmental Microbiology, Copenhagen University, Copenhagen, DK, Denmark

[2]Environmental Microbial Genomics (EMG), Aarhus University, Department of Environmental Science, Roskilde, DK, Denmark

*** Correspondence:**
Lars Hestbjerg Hansen
lhha@envs.au.dk

Supplementary Material

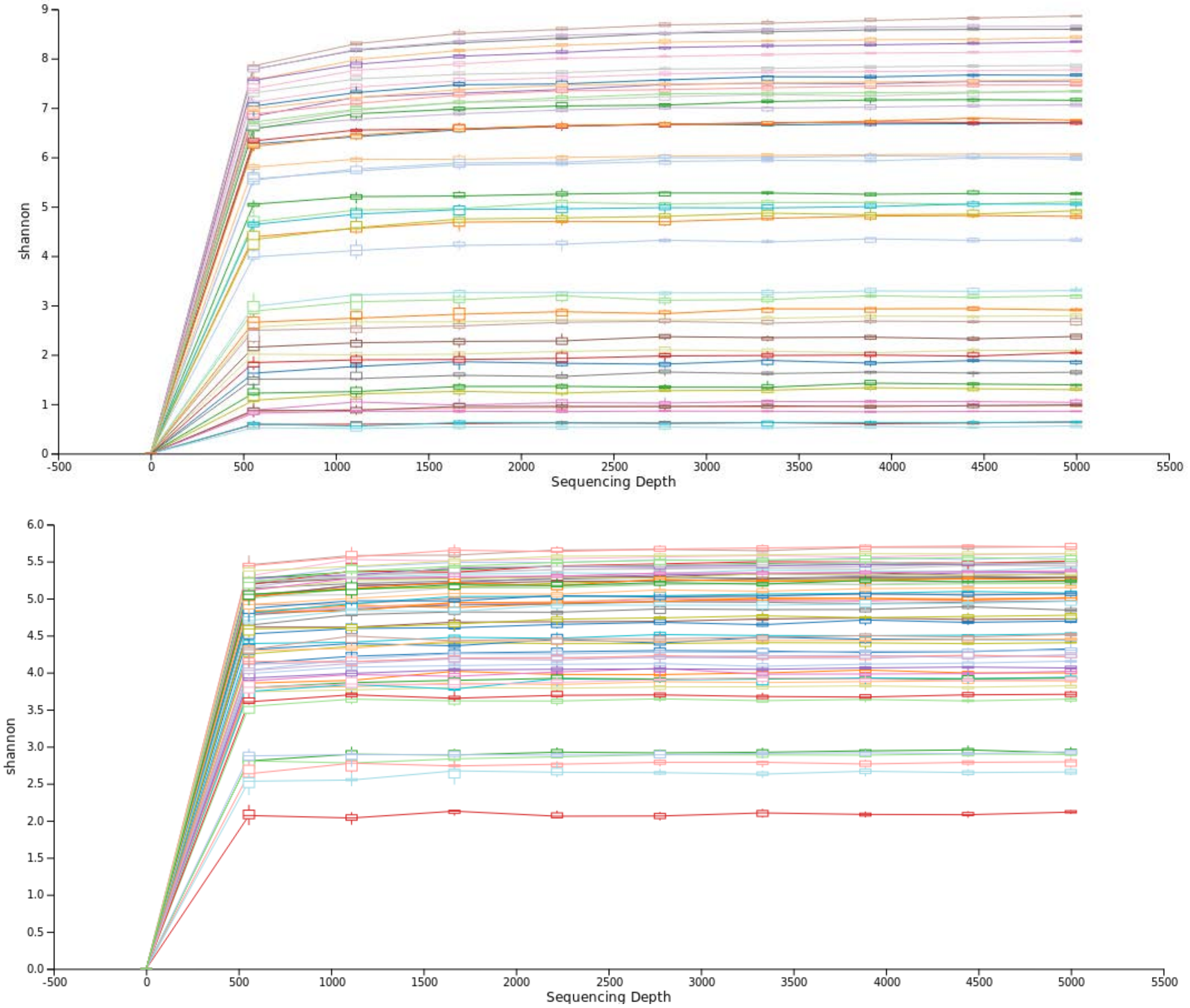

a

b

Figure S1: Rarefaction curves based on Shannon Index. Rarefaction curves obtained using 5000 reads from each samples and 10 iterations. a) all the 16S samples. b) All the ITS samples.

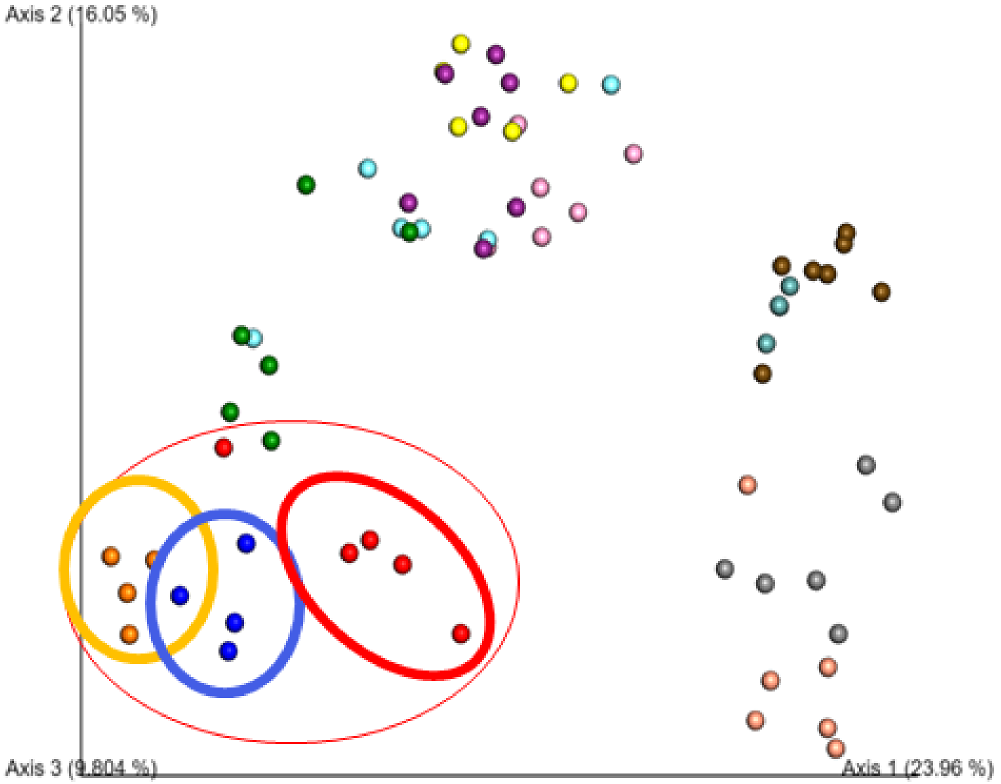

Figure S2; PCOA plot on ITS samples coloured by sampling day. In the thin-red circle you can see the samples collected during September in 3 different moment; The smaller but thicker circles include the replicates that were sequenced from the same collection date. As reported from Table 1 the three days were 15th (orange), 26th (blue) and 29th (red) of September.

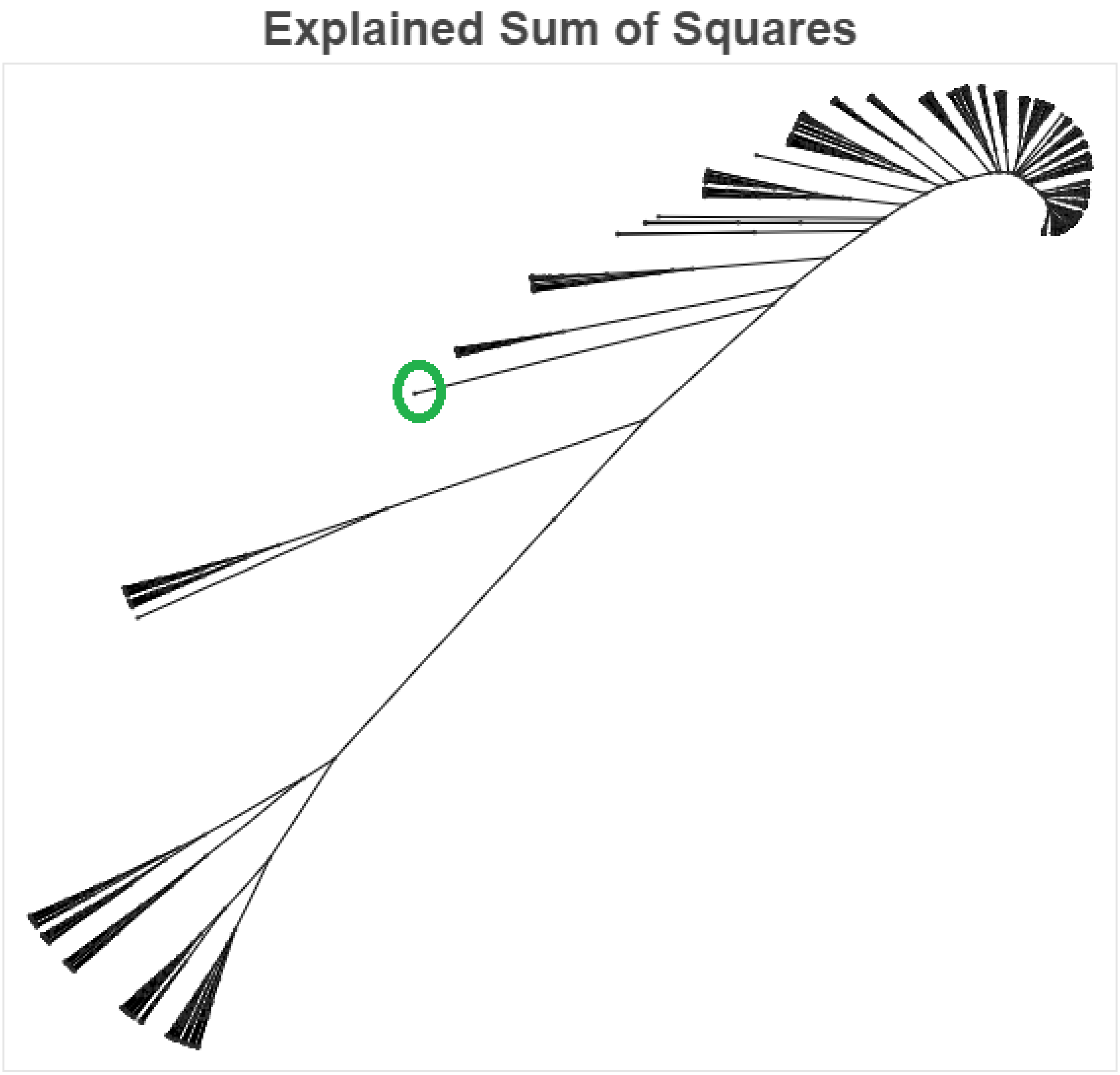

Figure S3; Gneiss balances tree built on the different groups of taxa in the 16S database; The model is obtained through a multivariate response linear regression. When several taxa branches out together it means they appear to belong to the same microbial niche. The green circle identify a single-taxon branch which belong to *Lactobacillacea*. Since it appears alone we assume there are no statistically relevant interaction with other members of the bacterial community. Furthermore it is considered to be differentially abundant between treatments with a p-value of 2.67*10^-9.

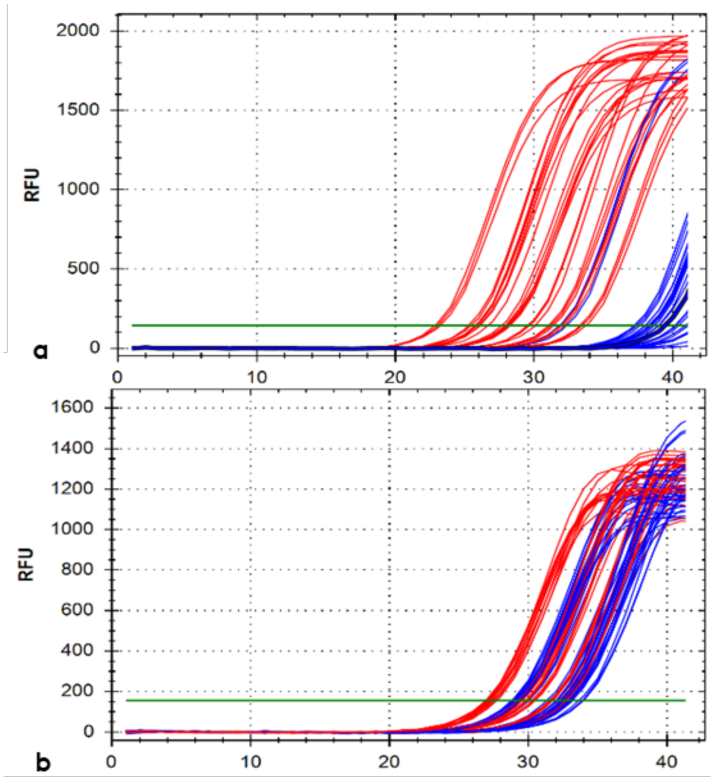

Figure S4; qPCR amplification results; a) amplification curves of MW-1 colored by treatment; in red biocontrol treated leaves, in blue copper treated leaves. b) amplification curves for fungal community colored by treatment; in read biocontrol treated samples and in blue copper treated leaves.
